# Y-Co metal-organic framework for sensitive electrochemical determination of doxorubicin hydrochloride

**DOI:** 10.5599/admet.3141

**Published:** 2025-12-18

**Authors:** Somayeh Mir, Niloufar Akbarzadeh Torbati, Vahid Amani, Somayeh Tajik, Hadi Beitollahi

**Affiliations:** 1 Department of Chemistry, University of Sistan and Baluchestan, P.O. Box 98135-674, Zahedan, Iran; 2 Department of Chemistry Education, Farhangian University, P.O. Box 14665-889, Tehran, Iran; 3 Research Center of Tropical and Infectious Diseases, Kerman University of Medical Sciences, Kerman, Iran; 4 Environment Department, Institute of Science and High Technology and Environmental Sciences, Graduate University of Advanced Technology, Kerman, Iran

**Keywords:** Pharmaceutical formulations, chemotherapy treatment, electrochemical sensor

## Abstract

**Background and purpose:**

Cancer represents a major challenge to public health; therefore, identifying doxorubicin hydrochloride (DOX·HCl) as an important chemotherapy drug holds considerable significance.

**Experimental approach:**

An electrochemical sensing strategy was designed for DOX·HCl determination by using Y-Co bimetallic metal-organic framework (Y-Co MOF) modified carbon paste electrode (CPE). The Y-Co-MOF was successfully prepared via the solvothermal method.

**Key results:**

Characterizations using field emission scanning electron microscopy, transmission electron microscopy, X-ray diffraction, Fourier transform infrared spectroscopy and energy-dispersive X-ray spectroscopy with elemental mapping images were used to evaluate the morphological features, crystalline structure, functional groups, and elemental composition of the Y-Co-MOF. From cyclic voltammetric studies, well-defined redox peaks of DOX·HCl with improved response peak currents at lower overpotentials were observed on the surface of Y-Co MOF/CPE compared to the unmodified CPE. This indicated that the as-prepared modified CPE has a strong and efficient redox capability toward DOX·HCl. Under the optimized parameters and conditions, the linear response range from differential pulse voltammetry measurements for DOX·HCl were 0.0025 to 100.0 μM, with a low limit of detection of 0.001 μM. Finally, the ability of the designed sensing platform to determine the amounts of DOX·HCl in the injection sample was studied and it has been observed a high and efficient ability with satisfactory values of recovery and relative standard deviation.

**Conclusion:**

This analytical approach offers a useful means for the analysis of pharmaceutical formulations, providing potential advantages in cancer therapy.

## Introduction

Cancer refers to a diverse group of diseases characterized by the uncontrolled growth and division of abnormal cells. These cells not only multiply rapidly but also invade surrounding tissues and spread to other parts of the body, leading to damage and disruption of normal bodily functions [1]. Chemotherapy is a commonly used cancer treatment that involves chemical drugs to target cancer cells by inhibiting or slowing their growth [2]. These agents exert their therapeutic effects primarily by causing DNA damage. These chemotherapeutic agents disrupt DNA replication and cell division by inducing DNA damage. Despite the effectiveness of chemotherapy in treating cancer, it can affect healthy cells, which is one of the main consequences of chemotherapy [3]. Furthermore, this treatment method also leads to numerous side effects [3]. Therefore, accurate detection and quantification of chemotherapeutic drugs are essential to ensure effective treatment while reducing adverse effects. Doxorubicin belongs to the anthracycline family of antibiotics [4,5]. Doxorubicin exerts its action through two proposed mechanisms [6]. It primarily operates by intercalating into DNA, inhibiting the activity of the topoisomerase II enzyme. This inhibition impairs DNA replication and transcription, ultimately leading to cell death. Furthermore, doxorubicin generates free radicals, causing DNA damage and oxidative stress, thereby enhancing cell death. These mechanisms contribute to doxorubicin's anticancer effects. Although doxorubicin has shown significant effectiveness in treating cancer, it is accompanied by several potential side effects. A significant side effect of doxorubicin is its potential to induce cardiotoxicity, leading to irreversible damage to heart muscle (myocardium) [7]. Other side effects of doxorubicin include vomiting, nausea, pain, hair loss, emergence of drug resistance, and increased susceptibility to infections [8]. So far, various studies have been conducted to develop methods for the determination of doxorubicin hydrochloride (DOX·HCl) in different samples. The standard methods are based on high-performance liquid chromatography (HPLC) [9], liquid chromatography-mass spectrometry (LC-MS) [10], capillary electrophoresis [11], chemiluminescence [12], spectrophotometry [13] and electrochemistry [14-16]. Although each employed method has distinct characteristics, electrochemical-based analytical methods, especially electrochemical sensors, remain an efficient alternative due to their fast response time, low cost, high sensitivity and portability [17-23]. A major challenge in the voltammetric determination of electroactive compounds using bare electrodes is the slow redox kinetics associated with their electrochemical reactions and electrode surface contamination, particularly for analysing complex samples. Recently, it has been reported that the sensitivity of electrochemical sensors can be enhanced by coating active compounds onto the surface of unmodified electrodes (electrode modification) for the determination of target compounds [24-29].

Nanotechnology is an emerging technology with vast potential across science and industry. Its ability to manipulate matter at the nanoscale opens new possibilities for innovation in electronics, medicine, environment, energy, and other fields [30-38]. In particular, nanostructured materials with good electrocatalytic activity, high surface-area-to-volume ratios, high adsorption capacities, large numbers of active sites, and simple surface modifications have shown promise for the design of efficient electrochemical sensors [39-44]. Metal-organic frameworks (MOFs) represent a class of highly porous materials composed of metal ions or clusters coordinated to organic linkers, which have attracted significant attention of researchers over the past few decades. MOFs, owing to their high structural tunability, greater number of active sites, high surface areas, customizable pore sizes, and other properties, have found widespread applications in diverse fields, including catalysis, sensing, energy, gas storage and separation, and biomedical applications [45-49]. Additionally, improvements in the catalytic capabilities of MOFs have attracted significant attention and have effectively expanded their applications. Bimetallic MOFs can significantly enhance the catalytic activity in electrochemical applications compared to monometallic compounds [50,51].

This work aimed to design and construct a new, straightforward electrochemical sensing platform for the determination of DOX·HCl. Y-Co-MOF modified CPE was applied as a working electrode for DOX·HCl determination based on the differential pulse voltammetry (DPV) method. Initial studies using cyclic voltammetry (CV) demonstrated that the presence of Y-Co-MOF in the carbon paste composition can significantly enhance the redox peak currents of DOX·HCl and shift their redox potentials to lower values. The Y-Co-MOF/CPE demonstrated a linear current response with the concentrations of DOX·HCl in the range of 0.0025 to 100.0 μM. Also, it showed a low detection limit (LOD) of 0.001 μM. The application of the Y-Co-MOF/CPE was indicated by determining DOX·HCl in the injection sample with satisfactory results.

## Experimental

### Apparatus and chemicals

The FT-IR spectrum was obtained by using a Shimadzu-470 spectrometer with a KBr disk, covering the spectral range from 4000 to 400 cm^-1^. The XRD data were collected using an expert diffractometer, the Advance D8 from Germany, utilizing copper Kα radiation (*λ* = 0.15406 nm). The FE-SEM images were obtained by the EM8000F scanning electron microscope (Kyky, China) attached with an EDS spectrometer. The transmission electron microscope (TEM) analysis was performed using the Philips em208s, 100 kV. The cyclic voltammetric, chronoamperometric and differential pulse voltammetric responses were recorded using a conventional three-electrode electrochemical system on an Autolab PGSTAT302N electrochemical workstation (Metrohm, Utrecht, Netherlands). In this electrochemical system, Ag/AgCl/KCl (3.0 M) was applied as the reference electrode (RE), Pt wire was applied as the counter electrode (CE), and unmodified CPE or Y-Co-MOF modified CPE were applied as the working electrodes (WEs). All the solvents and chemicals were utilized in analytical grade without purification. They were purchased from Merck and Sigma-Aldrich companies.

### Synthesis of Y-Co bimetallic metal-organic frameworks

Y-Co bimetallic metal-organic frameworks (Y-Co-MOF) were prepared via solvothermal synthesis. In the process, 0.3 mmol of Co(NO_3_)_2_·6H_2_O (0.1 g) and 0.7 mmol of Y(NO_3_)_3_·6H_2_O (0.3 g) were dissolved in N,N-dimethylformamide (DMF) (18 mL) under ultrasonication to create a homogeneous solution. Subsequently, 2.0 mmol (0.4 g) of 1,3,5-benzene tricarboxylic acid (BTC) was dissolved in 18 mL of DMF, and the resulting solution was slowly added to the metal precursor solution and stirred for 30 minutes to ensure thorough blending. The final solution was then transferred to a Teflon-lined stainless-steel autoclave and heated at 120 °C for 48 hours. Once the autoclave was allowed to cool naturally to room temperature, the precipitated bimetallic Y-Co-MOF was separated by centrifugation. The collected product was washed several times with DMF and then dried in an oven at 100 °C for 24 hours.

### Carbon paste electrode preparation and modification

To prepare Y-Co-MOF modified CPE, 0.485 g of graphite powder and 0.015 g of Y-Co-MOF were placed in a mortar and mixed well. Then, several drops of paraffin oil were added to the above mixture, followed by thorough grinding for about 30 min, yielding a uniform, homogeneous carbon paste. A portion of carbon paste was carefully packed into the cavity of a 3 mm internal-diameter glass tube to a depth of approximately 5 mm. Electrical contact with the paste was established by inserting a copper (Cu) wire into the tube, ensuring a conductive connection. Finally, a shiny and smooth surface was obtained by extruding the carbon paste from the glass tube, followed by careful polishing on a flat paper. To compare the electrocatalytic performance of Y-Co-MOF/CPE with that of CPE without Y-Co-MOF, the unmodified CPE was prepared using the same procedure.

## Results and discussion

### Characterization of Y-Co bimetallic metal-organic frameworks

**Figure 1.**
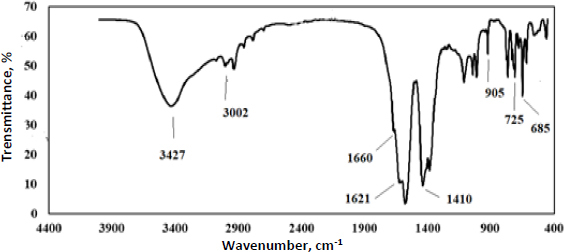
FT-IR spectrum of Y-Co-MOF

The Fourier transform infrared (FTIR) spectrum of Y-Co-MOF is exhibited in Figure 1. The results indicate that the C-H vibrational modes correspond to absorption bands observed in the range of 2800 to 3100 cm^-1^. Meanwhile, a broad peak attributed to O-H vibrations appears at 3427 cm^-1^. The peaks located at 685 and 725 cm^-1^ are probably related to the stretching vibrations of the Y-O bands and Co-O bands [52,53]. The observed peaks at 1660 cm^-1^ and 905 cm^-1^ were attributed to the C=C vibration of the aromatic ring and the C-H vibration, respectively. The presence of symmetric and asymmetric stretching vibrations of carboxyl groups (COOH) at 1410 and 1621 cm^-1^ confirmed the formation of the MOF [54,55].

**Figure 2.**
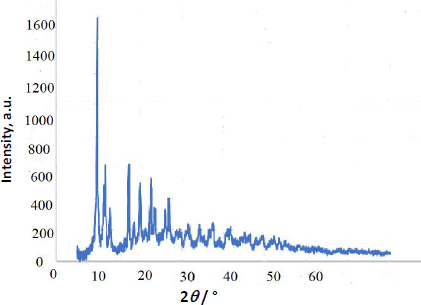
XRD pattern of the Y-Co-MOF

Figure 2 shows the X-ray diffraction (XRD) pattern of the Y-Co-MOF nanostructure. The diffraction peaks of Y-Co-MOF are identified at 2*θ* = 9.69, 11.29, 11.74, 17.33, 18.57, 20.09, 21.25, 22.67, 23.57, 26.11, 26.93, 31.67, 34.31, 36.68 and 37.75°. The diffraction pattern of Y-Co-MOF is similar to and consistent with those of Y-MOF and Co-MOF [55], confirming the structural data for Y-Co-MOF.

**Figure 3.**
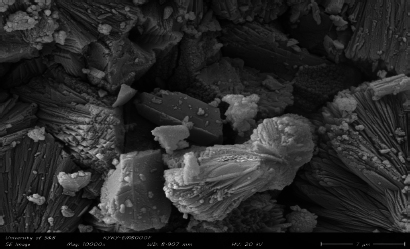
FE-SEM image of Y-Co-MOF

**Figure 4.**
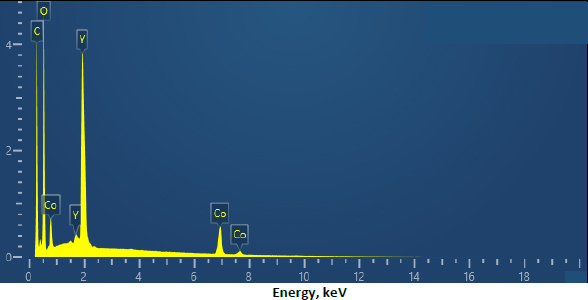
EDX analysis of Y-Co-MOF.

**Figure 5.**
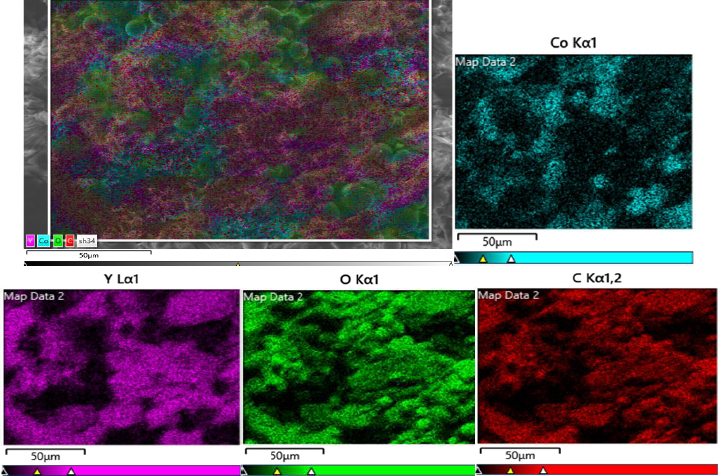
The elemental mapping images of Y-Co-MOF

**Figure 6.**
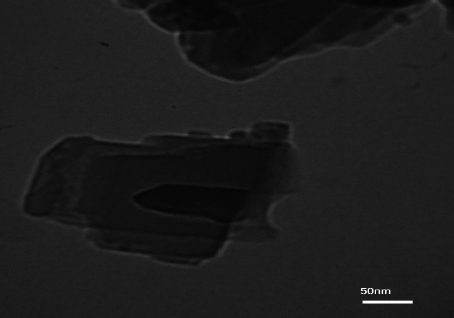
TEM image of Y-Co-MOF

A field-emission scanning electron microscopy (FE-SEM) image of the Y-Co-MOF is shown in Figure 3. From the FE-SEM image, this nanostructure exhibits a two-dimensional morphology, with interconnected, closely packed nanosheets. Energy-dispersive X-ray spectroscopy (EDX) analysis reveals the elemental composition of Y-Co-MOF. From the EDX spectrum, Y-Co-MOF consists of Co, Y, C and O elements (Figure 4). Also, the elemental mapping images were recorded to show the homogeneous distribution of constituent elements across the Y-Co-MOF (Figure 5). The TEM image of Y-Co-MOF highlights a two-dimensional nanosheet array with a clearly defined thin, sheet-like structure (Figure 6).

### Evaluating the CV responses of unmodified CPE and Y-Co-MOF/CPE for DOX·HCl determination

To evaluate the influence of the pH of electrolyte solution on the oxidation of DOX·HCl on the Y-Co-MOF/ /CPE, the DPVs were recorded in PBS (0.1 M) at different values of pH (from pH 3.0 to pH 9.0). Based on DPV measurements, the peak currents associated with the oxidation of DOX·HCl increased with increasing pH until pH 7.0, after which they decreased. Therefore, the high response peak current was observed at pH 7.0 of PBS. Hence, PBS 0.1 M with pH 7.0 was chosen as the optimal condition for further experiments.

**Figure 7.**
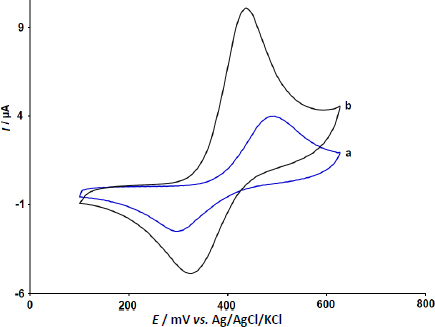
CVs for (a) unmodified CPE and (b) Y-Co-MOF/CPE in PBS in the presence of 20.0 μM DOX·HCl; scan rate 50 mV s^-1^

The electrochemical behaviour of DOX·HCl at unmodified and modified CPEs was evaluated by using the CV method. Figure 7 exhibits the CV responses of unmodified CPE (cyclic voltammogram a) and Y-Co-MOF/CPE (cyclic voltammogram b) in PBS containing 20.0 μM DOX·HCl recorded at the scan rate of 50 mV s^-1^.

From the recorded CVs, quasi-reversible redox behaviour of DOX·HCl was observed at the surfaces of both electrodes. The oxidation and reduction peaks of DOX·HCl with low currents (anodic peak current: *I*_pa_ = 3.9 μA and cathodic peak current: *I*_pc_ = -2.5 μA) were observed at 500 mV (anodic peak potential - *E*_pa_) and 300 mV (cathodic peak potential - *E*_pc_), respectively, indicating a slow rate of electron transfer at the unmodified CPE. However, by modifying CPE with Y-Co-MOF, the electrode response for DOX·HCl determination increased significantly.

For Y-Co-MOF modified CPE, a pair of strong and well-defined redox peaks with improved currents (*I*_pa_ = 10.0 μA and *I*_pc_ = -4.8 μA) were observed at *E*_pa_ = 435 mV and *E*_pc_ = 330 mV. Also, the peak-to-peak separation decreased from 200 mV at unmodified CPE to 105 mV in the case of Y-Co-MOF/CPE. Therefore, the Y-Co-MOF/CPE has higher electrocatalytic activity towards the redox process of DOX·HCl due to the presence of Y-Co-MOF in the composition of carbon paste, which can act as an effective material to improve the rate of electron transfer.

### Influence of potential scan rate on the response of Y-Co-MOF/CPE

**Figure 8.**
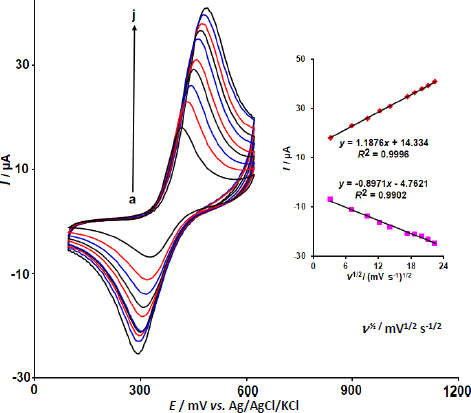
CVs recorded at Y-Co-MOF/CPE in PBS containing 50.0 μM DOX·HCl at different scan rates (10, 50, 100, 150, 200, 300, 350, 400, 450 and 500 mV s^-1^). Inset: Linear dependence between redox peak currents of DOX·HCl and *ν*^1/2^

The effect of the potential scan rate on the redox process of DOX·HCl (50.0 μM) in PBS was investigated by CV at the surface of Y-Co-MOF/CPE, with scan rates ranging from 10 to 500 mV s^-1^ (Figure 8). As the scan rate increased, the currents of the redox peaks associated with the redox process of DOX·HCl increased. Also, the redox peak currents increase in proportion to the square root of the scan rate (*ν*^1/2^), demonstrating that a diffusion-controlled reaction occurred, as shown in Figure 8 (Inset).

### Chronoamperometric investigations

**Figure 9.**
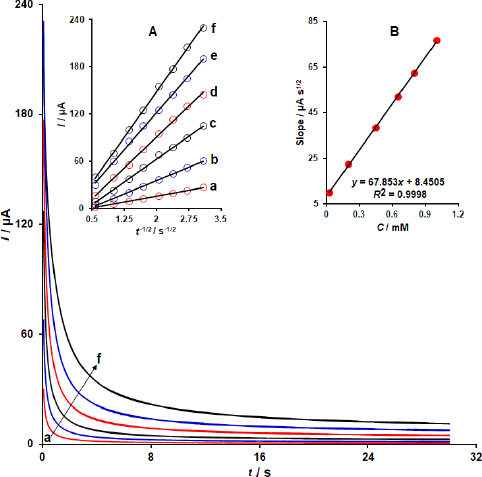
Chronoamperometric responses of Y-Co-MOF/CPE towards different concentrations (0.025, 0.2, 0.45, 0.65, 0.8 and 1.0 mM) of DOX·HCl. Insets: (A) Cottrell plots obtained from chronoamperometric responses for each concentration of DOX·HCl) and (B) Linear relationship between the slope of the Cottrell plots and the concentration of DOX·HCl).



Estimation of the diffusion coefficient (*D*) for the electrochemical process of DOX·HCl at Y-Co-MOF/CPE was performed by the chronoamperometry method. The chronoamperograms were recorded by applying a potential of 460 mV vs. Ag/AgCl/KCl (3.0 M) at the working electrode for DOX·HCl at different concentrations (Figure 9). Diffusion coefficient can be estimated by using the Cottrell equation in diffusion-controlled conditions by employing Equation (1):

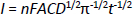

(1)


The Cottrell plots of current (*I*) *vs. t*^-1/2^ obtained from chronoamperograms recorded for different concentrations of DOX·HCl are illustrated in Figure 9, inset A. Moreover, the slopes of the Cottrell plots were plotted against the concentration of DOX·HCl, yielding a linear relationship (Figure 9, Inset B). The diffusion coefficient for DOX·HCl was estimated to be 4.78×10^-5^ cm^2^ s^-1^.

### Quantification of DOX·HCl based on DPV measurements

**Figure 10.**
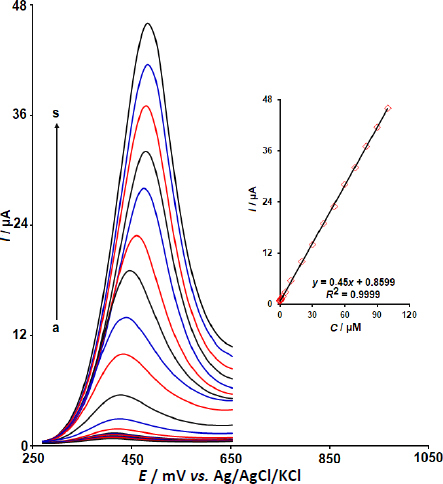
DPV responses of Y-Co-MOF/CPE toward various concentration (0.0025, 0.01, 0.1, 0.25, 0.5, 0.75, 1.0, 2.5, 5.0, 10.0, 20.0, 30.0, 40.0, 50.0, 60.0, 70.0, 80.0, 90.0 and 100.0 μM) of DOX·HCl in PBS (DPVs were recorded at the following conditions: scan rate of 50 mV/s, step potential of 0.01 V, and pulse amplitude of 0.025 V). Inset: Corresponding linear relationship between *I*_pa_ and concentration of DOX·HCl

To quantify DOX·HCl, we used DPV. This technique aims to reduce the effect of background capacitive current, thereby enhancing the prepared sensor's sensitivity and signal-to-noise ratio. Figure 10 shows the DPVs recorded at Y-Co-MOF/CPE in PBS at different DOX·HCl concentrations.

The DPV measurements show that *I*_pa_ increases gradually with increasing DOX·HCl concentration. Also, using DPV observations for various concentrations of DOX·HCl in the range of 0.0025 to 100.0 μM, the linearity was described by the following equation: *I*_pa_ = 0.45*C*_DOX_·_HCl_ + 0.8599 (*R*^2^ = 0.9999). The corresponding calibration plot is shown in the inset of Figure 10. The LOD was calculated by using Equation (2):



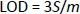

(2)


where *m* is the slope of the calibration plot and *S* is the standard deviation (*n* = 12) of the DPV currents corresponding to the blank solution. The LOD value for DOX·HCl was calculated to be 0.001 μM.

### Injection sample analysis

Finally, the designed sensing platform was used to assess the applicability of the proposed method for determining DOX·HCl in the injection sample. The diluted injection sample was analyzed by DPV five times. Then, the injection samples were spiked with various concentrations of DOX·HCl and analyzed by DPV. Based on DPV responses, the designed sensing platform showed high recovery values of 97.1 to 103.8 % for DOX·HCl analysis, and the relative standard deviation (RSD) for five measurements per sample was less than 3.5 % (Table 1). Therefore, the Y-Co-MOF/CPE electrochemical sensor shows strong potential for the determination of DOX·HCl in pharmaceutical formulations.

**Table 1.** Results obtained from the determination of DOX·HCl in the injection sample using Y-Co-MOF/CPE. All the experiments were carried out five times (*n* = 5)

Concentrations of DOX·HCl, μM		Recovery, %	RSD, %
Spiked	Found		
0	2.9	-	3.5
2.0	5.0	102.0	2.2
3.0	5.8	98.3	2.9
4.0	6.7	97.1	3.3
5.0	8.2	103.8	1.8

## Conclusion

In this study, Y-Co-MOF was used to modify the CPE via a simple method to increase its active surface area and was applied as a sensing platform for the determination of DOX·HCl. The electrochemical behaviour of DOX·HCl was evaluated using the CV method. The Y-Co-MOF-modified CPE shows improved electrocatalytic performance for the redox process of DOX·HCl. Such improvement is related to the use of Y-Co-MOF in the composition of carbon paste, as well as its beneficial properties. The quantitative measurements by the DPV method provide a linear response with the concentration range from 0.0025 to 100.0 μM, with a low LOD of 0.001 μM. Additionally, the Y-Co-MOF/CPE showed potential for the determination of DOX·HCl in the injection sample.
